# Clinical Aspects of IgG4-Related Orbital Inflammation in a Case Series of Ocular Adnexal Lymphoproliferative Disorders

**DOI:** 10.1155/2012/635473

**Published:** 2012-04-02

**Authors:** Masayuki Takahira, Yoshiaki Ozawa, Mitsuhiro Kawano, Yoh Zen, Shoko Hamaoka, Kazunori Yamada, Kazuhisa Sugiyama

**Affiliations:** ^1^Department of Ophthalmology, Kanazawa University, 13-1 Takara-machi, Kanazawa, Ishikawa 9208640, Japan; ^2^Division of Rheumatology, Kanazawa University, Kanazawa, Ishikawa 9208640, Japan; ^3^Institute of Liver Studies, King's College Hospital, London, SE5 9RS, UK

## Abstract

The most frequent ocular adnexal tumors and simulating lesions are lymphoproliferative disorders (LPDs), including malignant lymphomas and orbital inflammation with lymphoid hyperplasia or infiltration. IgG4-related orbital inflammation (IgG4-ROI) often involves lacrimal glands and other orbital tissues and is an important differential diagnosis. The present study evaluated clinical aspects of IgG4-ROI in a case series of orbital LPD. Sixty-two consecutive cases of orbital LPD, pathologically diagnosed from November, 2004, through March, 2011, were investigated. Histological types were 22 cases with MALT lymphoma, 11 cases with diffuse large B-cell lymphoma (DLBCL), 3 cases with other malignant lymphomas, 16 cases with IgG4-ROI, and 10 cases with non-IgG4-ROI. Ages of the IgG4-ROI group (56 ± 10 yrs) were significantly lower than the MALT lymphoma (71 ± 12 yrs) and DLBCL (75 ± 14 yrs) groups. Orbital lesions other than lacrimal glands were present in six cases including extraocular muscle swelling, mass lesions surrounding the optic nerve, and supraorbital and infraorbital nerves enlargements. Although none of the malignant lymphomas were related to IgG4, previous evidence suggested that malignant lymphomas can arise from IgG4-ROI. Based on this study (26%) and another report (33%), it is likely that nearly a quarter of orbital LPD are IgG4-ROI.

## 1. Introduction

The most frequent tumors and simulating lesions in ocular adnexa are lymphoproliferative disorders (LPDs), including malignant lymphomas and orbital inflammation with lymphoid hyperplasia or infiltration, some of which are historically called “orbital pseudotumors.” In Japanese studies, Goto [1] reported that the rate of LPD among 409 cases with orbital tumors and simulating lesions was 43%, while Ohtsuka et al. [2] described that the rate was 49% out of 213 cases. In the United States, LPD is a common orbital disease although the rate seems to be somewhat lower: 24% of LPD out of 703 cases with orbital lesions was reported by Shields et al. [3], and 26% of LPD out of 268 cases was reported by Shinder et al. [4]. 

When patients with suspected orbital LPD are encountered, tissue biopsy is preferred since image examinations alone cannot distinguish inflammatory lesions from malignant lymphomas. Pathological examination can also detect whether the lesion is related to IgG4 or not. IgG4-related disease (IgG4-RD) often involves lacrimal glands, which is now known as IgG4-related Mikulicz's disease [5, 6] or IgG4-related dacryoadenitis [7, 8] by many recent reports over several years. Recently, it was also elucidated that IgG4-related orbital inflammatory lesions include other ocular adnexal tissues such as extraocular muscles [9] and periorbital membrane [10]. Therefore, IgG4-RD is a differential diagnosis in orbital LPD. The question is then raised as to what percentage of orbital LPD is related to IgG4. In this study, an orbital LPD case series was investigated and clinical aspects of IgG4-related orbital lymphoproliferative disorders were evaluated.

## 2. Patients and Methods

In Kanazawa University Hospital in Japan, a 47-year-old woman with IgG4-immunopositive histopathology and an elevated serum IgG4 level of 1000 mg/dL was diagnosed as a first case of IgG4-related dacryoadenitis in November, 2004 [8]. From that time through March, 2011, sixty-two cases (27 men and 35 women; mean age, 66 ± 14 yrs; range 32–89 yrs) were pathologically diagnosed with orbital lymphoproliferative diseases (LPD) from surgical samples of ocular adnexal tissue. The two main categories of orbital LPD were malignant lymphomas and orbital inflammations: the latter included reactive lymphoid hyperplasia, lymphoid infiltrated lesions, and inflammatory pseudotumor. This consecutive 62 case series was investigated retrospectively. Conjunctival lesions were not enrolled in this study because conjunctival involvement in IgG4-RD has never been experienced in previous reports [9, 11] or in the author's institution. Intraocular lymphoma belongs to CNS lymphoma and thus was also excluded in this study. In most cases, immunoglobulin heavy chain gene rearrangement in surgical samples was also examined to support the differential diagnosis of malignant lymphoma. Diagnostic criterion for positive IgG4-immunostaining in orbital tissue (IgG4-related orbital disease) was either (1) the ratio of IgG4-positive cells to IgG-positive cells (IgG4+/IgG+ cells) was more than 40% [12], or (2) the number of IgG4-positive cells was more than 30 per high power microscopy field [13]. Mouse monoclonal antibody anti-human IgG4 (05-3800, ZYMED, USA) and rabbit polyclonal antibody anti-human IgG (A0423, Dako, USA) were used for immunostaining. Serum IgG and IgG4 were measured in all of the cases with an IgG4-positive pathological diagnosis. IgG4-related orbital lesions including lacrimal gland swelling, extraocular muscle enlargement, and other mass lesions were evaluated using computed tomography (CT) and/or magnetic resonance imaging (MRI).

## 3. Results

**Figure 1:**
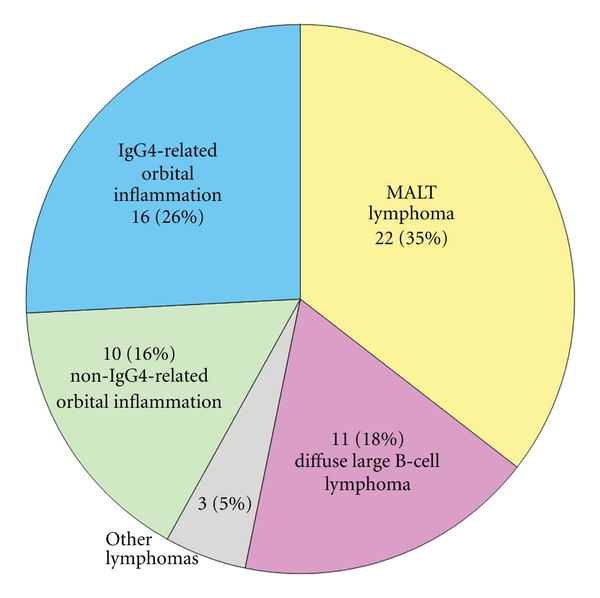
Breakdown of diagnosis in orbital lymphoproliferative disorders. Patients were a consecutive 62 case series with orbital lymphoproliferative disorders pathologically diagnosed from November, 2004, to March, 2011, in Kanazawa University Hospital, Japan. Cases with conjunctival and intraocular lesions were excluded. MALT lymphoma is an abbreviation for extranodal marginal zone B-cell lymphoma of mucosa-associated lymphoid tissue.

**Figure 2:**
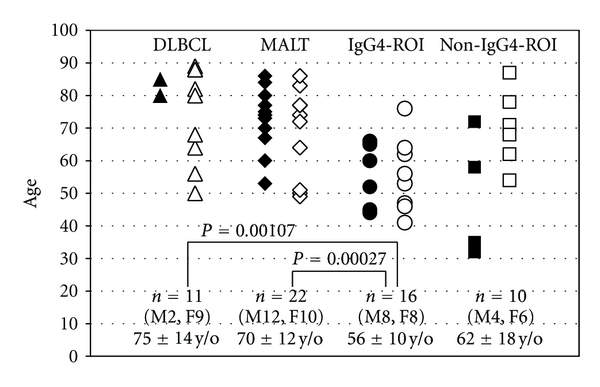
Ages of orbital lymphoproliferative disorders. Each plot depicts age at diagnosis in groups of MALT lymphoma, DLBCL (diffuse large B-cell lymphoma), IgG4-related orbital inflammation and non-IgG4-related orbital inflammation. Closed and open plots represent men and women, respectively.

Histological types of the 62 orbital LPD were 22 cases (35%) with extranodal marginal zone lymphoma of mucosa-associated lymphoid-tissue type (MALT lymphoma), 11 cases (18%) with diffuse large B-cell lymphoma (DLBCL), 3 cases (5%) with other malignant lymphomas (1 mantle cell lymphoma, 1 NK/T cell lymphoma, 1 small lymphocytic lymphoma), 16 cases (26%) with IgG4-related orbital inflammation (IgG4-ROI), and 10 cases (16%) with non-IgG4-related orbital inflammation (non-IgG4-ROI) (Figure 1). None of the malignant lymphomas showed a relationship with IgG4 in this case series.  Figure 2 depicts the ages of the four groups of DLBCL, MALT lymphoma, IgG4-ROI, and non-IgG4-ROI. Ages of the IgG4-ROI group averaged 56 ± 10 yrs, which was significantly lower than those of the MALT lymphoma (71 ± 12 yrs, *P* = 0.00027 in a Student's *t*-test) and DLBCL (75 ± 14 yrs, *P* = 0.00107) groups. There were no significant age differences between other combinations of the four groups.

**Figure 3:**
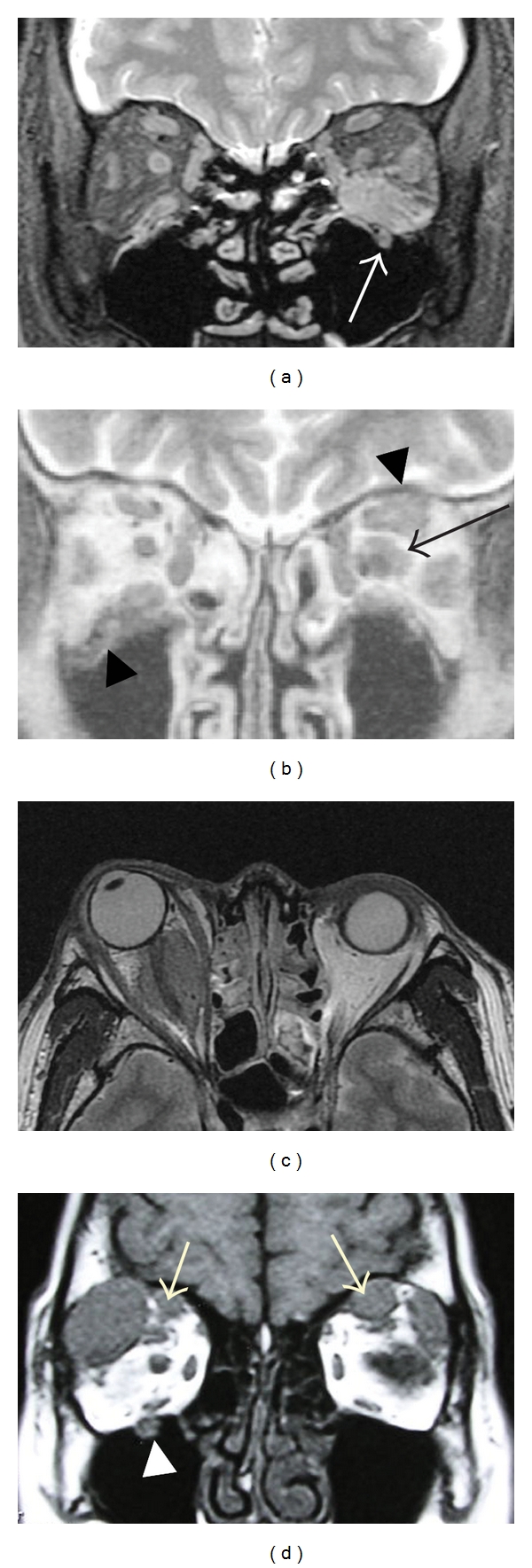
Representative cases with IgG4-related orbital inflammatory lesions other than lacrimal glands. (a) Enlargements in the left inferior rectus muscle and infraorbital nerve (arrow) in a 65-year-old man with a serum IgG4 of 404 mg/dL (case number 7 in Table 1). (b) Swelling of the left superior and lateral rectus muscles, a mass lesion around the left optic disc (arrow), and enlargements of the left supraorbital nerve and the right infraorbital nerve (arrow heads) were seen in a 60-year-old man with a serum IgG4 of 463 mg/dL (case number 8). (c) Mass lesion around the right optic disc was observed in a 44-year-old man with a serum IgG4 of 599 mg/dL (case number 11). (d) Bilateral supraorbital nerve enlargements (arrows) and right infraorbital nerve enlargement were observed in a 47-year-old woman with a serum IgG4 of 1000 mg/dL (case number 16). Magnetic resonance images are T1-weighted in (b) and (d) and T2-weighted in (a) and (c).

Clinical data of sixteen cases of IgG4-ROI are summarized in Table 1, which were sorted in ascending order of serum IgG4 level. The age ranged between 41 to 76 yrs (mean ± SD;  56 ± 10  yrs, median; 58 yrs), and there was no sex difference (8 men and 8 women). These cases except one (number 1, detailed in the discussion) were accompanied by elevated serum IgG4 concentration (549 ± 293 mg/dL, *n* = 15, normal range < 135 mg/dL) and an increased ratio of serum IgG4/IgG (28 ± 12%, *n* = 15, normal range < 7%). In 9 patients, the serum IgE was also elevated. In all 16 cases, diagnosis was made by lacrimal gland biopsy. In pathology, these were characterized by lymphoplasmacytic infiltration, forming lymphoid follicles (germinal center) and sclerosing fibrosis [8]. IgG4-positive plasma cells were observed around lymphoid follicles and in intraglandular areas [8]. Orbital lesions other than lacrimal glands were detected by MRI in six cases (cases numbers 4, 7, 8, 11, 14, and 16 in Table 1) as shown representatively in Figure 3. Swollen extraocular muscles were seen in four cases (numbers 4, 7, 8 and 14). Supraorbital nerve (frontal nerve), and/or infraorbital nerve enlargements were observed in 4 cases (numbers 7, 8, 11, and 14). A mass lesion surrounding the optic nerve was detected in two patients (numbers 8 and 11). 

Steroid therapy was performed for all the IgG4-ROI cases except three patients because their eyelid swellings decreased in size after biopsy surgery (case numbers 1, 2, and 5) and because of normal serum IgG4 (case number 1) and diabetes mellitus (case number 2). Eleven cases underwent oral prednisolone tapering therapy with an initial dose of 20, 30, or 40 mg per day. In two cases with diabetes mellitus, lower doses of oral prednisolone (10 mg daily in case number 10 and 8 mg daily in case number 15) were administered initially and reduced to a maintenance dose. All of the 11 cases essentially responded well to initial doses of oral prednisolone, but in 5 cases (numbers 4, 8, 10, 11, and 14) ocular symptoms such as eyelid swelling deteriorated during tapering, and thus increase in the dosage was required. In case number 11 with retrobulbar mass (Figure 3(c)), oral prednisolone therapy alone (30 mg daily initially) failed to diminish his exophthalmos, and then intravenous methylprednisolone pulse (500 mg for 3 days) was additionally performed three times.

## 4. Discussion

In this study, none of the malignant lymphoma cases was related to IgG4. However, some previous reports suggested that orbital malignant lymphomas can be related to IgG4. Cheuk reported three cases of ocular adnexal lymphoma (2 MALT lymphoma and 1 follicular lymphoma) arising in IgG4-related dacryoadenitis, and that the rate of transformation of malignant lymphoma in the background of IgG4-ROI was approximately 10% [12]. Sato et al. first detected IgH gene rearrangement in two cases of ocular adnexal IgG4-related disease [11] and later reported seven patients with ocular adnexal MALT lymphoma arising from IgG4-related orbital disease [14]. On the other hand, he described a case of IgG4-producing MALT lymphoma of the lymph node [15]. Also in the orbital region, Oyama reported a case of IgG4-expressing MALT lymphoma in the lacrimal gland [16]. Based on these findings, there could be two possibilities of orbital MALT lymphoma arising from preexisting IgG4-ROI and de novo IgG4-positive orbital MALT lymphoma [12]. In any case, orbital biopsy should be mandatory to evaluate malignancy before treatment even if serological examination already detected elevated serum IgG4.

There is no doubt that IgG4-ROI most frequently involves the lacrimal gland, which is reported as IgG4-related dacryoadenitis and Mikulicz's disease [5–8]. In addition, lesions of IgG4-ROI other than the lacrimal glands were reported previously. Sato et al. reported that orbital masses other than the lacrimal gland were detected in 7 out of 21 cases with IgG4-ROI [11]. Kubota et al. reported a case with bilateral multiple extraocular muscle enlargement [9]. Mehta et al. presented a case of IgG4-ROI with enlargement of the infraorbital canal and periorbital membrane involvement [10]. Similar lesions were observed in the present case series of IgG4-ROI (Figure 3). In addition, mass lesions around the optic nerve were seen in two cases, indicating that involvement of the optic nerve sheath may not be rare in IgG4-ROI.

Among 16 cases of IgG4-ROI, one patient (case number 1) presented normal levels of serum IgG4. In this case, the ratio of IgG4+/IgG+ cells in immunohistochemistry was around 30%, somewhat lower than values in diagnostic criteria [6, 12], but IgG4-positive cells were more than 30 per high power microscopy field. The pathological findings that lymphoplasmacytic infiltration with germinal centers and dense sclerosing fibrosis was characteristic for IgG4-ROI. So far, we cannot resolve the reason for this discrepancy between pathological and serological findings. However, Kubota previously reported a patient with an inverse discrepancy who had negative findings on the IgG4 immunostaining despite presumably typical IgG4-ROI with elevated serum IgG4 [9].

The rate of IgG4-RD in 62 cases with orbital LPD was 26% in this study and was reported to be 33% out of 58 orbital LPD cases from another Japanese institute [17]. Thus, it is likely that nearly a quarter of orbital lymphoproliferative disorders are estimated to be related to IgG4. Further multicenter studies will be required to confirm this rate and to evaluate the frequency and location of IgG4-ROI other than the lacrimal glands.

## Figures and Tables

**Table 1:** Clinical data in 16 cases with IgG4-related orbital disease.

Case number	Age	Sex	Orbital lesions	Lesions other than orbit	Serum IgG (mg/dL)	Serum IgG4 (mg/dL)	IgG4/IgG (%)	IgG4 + cells /HPF	Serum IgE (IU/mL)	Therapy
1	62	F	LG	None	1690	29	2	43	14	None
2	66	M	LG	SG	1960	164	8	143	1621	None
3	56	F	LG	Lung lesion	1671	194	12	46	254	Steroid
4	46	F	LG, EOM	SG, retroperitoneal fibrosis	1350	209	15	92	170	Steroid
5	41	F	LG	Sinusitis	1554	260	17	62	178	None
6	53	F	LG	SG	2260	359	16	58	611	Steroid
7	65	M	LG, EOM, ION	Sinusitis, submandibular lymphadenopathy	1448	404	28	98	3374	Steroid
8	60	M	LG, EOM, OPN, SON, ION	SG, neck skin nodule	1220	463	38	82	4608	Steroid
9	64	F	LG	SG, neck lymph nodes TB	1820	486	27	83	90	Steroid
10	66	M	LG	SG, lung lesion, axillary lymphadenopathy	1900	575	30	48	151	Steroid
11	44	M	LG, OPN, ION	None	1322	599	45	63	351	Steroid
12	76	F	LG	Nephritis	2860	769	27	58	428	Steroid
13	60	M	LG	SG,	1952	886	45	48	575	Steroid
14	45	M	LG, EOM, ION	Lung lesions, sinusitis	2260	914	40	74	72	Steroid
15	52	M	LG	SG	3440	949	28	50	973	Steroid
16	47	F	LG, SON, ION	SG	2350	1000	43	55	183	Steroid

				Normal range	870–1700	<135	<7	<10	<250	

LG: lacrimal gland, EOM: extraocular muscle, OPN: optic nerve, SON: supraorbital nerve, ION: infraorbital nerve, SG: salivary gland.

## References

[1] Goto H, Goto H (2008). Review of ocular tumor. *Practical Ophthalmology*.

[2] Ohtsuka K, Hashimoto M, Suzuki Y (2005). A review of 244 orbital tumors in Japanese patients during a 21-year period: origins and locations. *Japanese Journal of Ophthalmology*.

[3] Shields JA, Shields CL, Scartozzi R (2004). Survey of 1264 patients with orbital tumors and simulating lesions: the 2002 Montgomery Lecture, part 1. *Ophthalmology*.

[4] Shinder R, Al-Zubidi N, Esmaeli B (2011). Survey of orbital tumors at a comprehensive cancer center in the United States. *Head and Neck*.

[5] Yamamoto M, Takahashi H, Sugai S, Imai K (2005). Clinical and pathological characteristics of Mikulicz’s disease (IgG4-related plasmacytic exocrinopathy). *Autoimmunity Reviews*.

[6] Masaki Y, Dong L, Kurose N (2009). Proposal for a new clinical entity, IgG4-positive multiorgan lymphoproliferative syndrome: analysis of 64 cases of IgG4-related disorders. *Annals of the Rheumatic Diseases*.

[7] Cheuk W, Yuen HKL, Chan JKC (2007). Chronic sclerosing dacryoadenitis: part of the spectrum of IgG4-related sclerosing disease?. *American Journal of Surgical Pathology*.

[8] Takahira M, Kawano M, Zen Y, Minato H, Yamada K, Sugiyama K (2007). IgG4-related chronic sclerosing dacryoadenitis. *Archives of Ophthalmology*.

[9] Kubota T, Moritani S, Katayama M, Terasaki H (2010). Ocular adnexal IgG4-related lymphoplasmacytic infiltrative disorder. *Archives of Ophthalmology*.

[10] Mehta M, Jakobiec F, Fay A (2009). Idiopathic fibroinflammatory disease of the face, eyelids, and periorbital membrane with immunoglobulin G4-positive plasma cells. *Archives of Pathology and Laboratory Medicine*.

[11] Sato Y, Ohshima KI, Ichimura K (2008). Ocular adnexal IgG4-related disease has uniform clinicopathology. *Pathology International*.

[12] Cheuk W, Yuen HKL, Chan ACL (2008). Ocular adnexal lymphoma associated with IgG4+ chronic sclerosing dacryoadenitis: a previously undescribed complication of IgG4-related sclerosing disease. *American Journal of Surgical Pathology*.

[13] Kamisawa T, Funata N, Hayashi Y (2003). Close relationship between autoimmune pancreatitis and multifocal fibrosclerosis. *Gut*.

[14] Sato Y, Notohara K, Kojima M, Takata K, Masaki Y, Yoshino T (2010). IgG4-related disease: historical overview and pathology of hematological disorders: review article. *Pathology International*.

[15] Sato Y, Takata K, Ichimura K (2008). IgG4-producing marginal zone B-cell lymphoma. *International Journal of Hematology*.

[16] Oyama T, Takizawa J, Nakamura N, Aoki S, Aizawa Y, Abe H (2011). Multifocal mucosa-associated lymphoid tissue lymphoma associated with IgG4-related disease: a case report. *Japanese Journal of Ophthalmology*.

[17] Ohshima KI (2008). Ocular adnexal IgG4-related disorders: do they overlap with Mikulicz’s disease or MALT lymphoma?. *Neuro-Ophthalmology Japan*.

